# Treatment of Diabetes by Salicin

**Published:** 1878-10

**Authors:** 


TREATMENT OF DIABETES BY SALICIN.—Dr. Augustus Muller publishes his treatment of diabetes by salicin and salicylic acid compounds, and concludes—

1. That salicylate of soda can stop the symptoms of diabetes, but the relief is only for a time.

2. That said symptoms disappear much more rapidly with large doses, as 14 to 16 grammes of salicin.

3. That when intoxication is noticed, the medicine should be stopped.

4. That salicylate of soda slightly affects the kidneys, even in large doses.

